# Revisiting aneuploidy profile of surgically retrieved spermatozoa by whole exome sequencing molecular karyotype

**DOI:** 10.1371/journal.pone.0210079

**Published:** 2019-01-04

**Authors:** Stephanie Cheung, Peter N. Schlegel, Zev Rosenwaks, Gianpiero D. Palermo

**Affiliations:** 1 The Ronald O. Perelman and Claudia Cohen Center for Reproductive Medicine, Weill Cornell Medicine, New York, New York, United States of America; 2 Department of Urology, Weill Cornell Medicine, New York, New York, United States of America; University Hospital of Münster, GERMANY

## Abstract

Previous studies, including our own, have reported that spermatozoa isolated from the testis have remarkably higher occurrence of aneuploidy once isolated from azoospermic men. This notion, however, did not translate into a lower pregnancy rate nor a greater proportion of miscarriages. Indeed, ICSI offspring generated from surgically retrieved gametes did not suffer from increased karyotypic aneuploidy than children generated from ejaculated specimens. In recent years, aneuploidy assessments on a larger number of cells and utilizing more chromosome probes have reported a progressive decrease in chromosomal aberrations in spermatozoa directly retrieved from the seminiferous tubules. In light of the availability of more accurate molecular genetic techniques, we have decided to challenge the notion that sampling epididymal and testicular tissues yields spermatozoa with higher incidence of aneuploidy than those retrieved in the ejaculate. In a retrospective manner, we have carried out an analysis by FISH with 9 chromosome probes on at least 1000 cells from the ejaculates of 87 consenting men and the specimens of 6 azoospermic men, while spermatozoa of fertile donors were used as control. Aneuploidy by FISH yielded 0.9% for the donor control but rose in the study group to 3.6% in the ejaculated, 1.2% for the epididymal, and 1.1% for testicular spermatozoa. There were no differences in autosomal or gonosomal disomies, nor nullisomies. In this group, once the specimens of these men were used for ICSI, ejaculated spermatozoa yielded a 22% clinical pregnancy rate that resulted in 62.5% pregnancy loss. The surgically retrieved specimens yielded a 50% clinical pregnancy rate that progressed to term. To confirm our findings, in a prospective analysis, DNA sequencing was carried out on the ejaculates and surgical samples of 22 men with various spermatogenic characteristics. In this comparison, the findings were similar with actually a higher incidence of aneuploidy in the ejaculated spermatozoa (n = 16) compared to those surgically retrieved (n = 6) (*P*<0.0001). For this group, the clinical pregnancy rate for the ejaculated specimens was 47.2% with 29.4% pregnancy loss, while the surgically retrieved yielded a 50% clinical pregnancy rate, all progressing to term. A subsequent prospective combined assessment on ejaculated and surgically retrieved spermatozoa by FISH and NGS was performed on non-azoospermic men with high DNA fragmentation in their ejaculate. The assessment by FISH evidenced 2.8% chromosomal defects in the ejaculated and 1.2% in testicular biopsies while by NGS became 8.4% and 1.3% (*P* = 0.02), respectively. Interestingly, we evidenced a pregnancy rate of 0% with ejaculated while 100% with the testicular spermatozoa in this latter group. This indicates that improved techniques for assessing sperm aneuploidy on a wider number of cells disproves earlier reports and corroborates the safe utilization of testicular spermatozoa with a positive impact on chances of pregnancy.

## Introduction

Intracytoplasmic sperm injection (ICSI) is widely considered as the most effective treatment for male factor infertility, and it enables even azoospermic men to father their own child [1]. However, qualms still exist related to the invasiveness of the procedure and, especially, about the possibility of carrying and transmitting to offspring genetic defects related to male factor infertility. Therefore, it is important to have a proper anamnestic and physical evaluation of the male partner by the reproductive physician or urologist [2]. This assessment begins with a semen analysis, and once the patient is considered oligospermic or azoospermic, it is often supplemented by a peripheral karyotype or the detection of micro DNA deletions of the Y chromosome. Interestingly, among all semen parameters, the incidence of aneuploidy and specifically Y chromosomal defects seem to follow an inverse correlation with the sperm concentration [3, 4].

The most important factor in the chromosomal health of an embryo is the oocyte’s age. However, in an attempt to grant a competent embryo, this evaluation can be extended to the male gamete genome, particularly in the case of implantation failure in spite of a young female partner. It is important to re-evaluate the chromosomal complement of the spermatozoon generally considered at low risk for aneuploidy. A karyotype assessment of the spermatozoon with abnormal results assumes relevance particularly in cases with adequate sperm parameters and, most significantly, in male partners of couples with a reproductive history characterized by recurrent pregnancy loss with an aneuploid conceptus, in spite of the absence of advanced maternal age. Targeted genetic screening is also appropriate for azoospermic men known to have a higher incidence of constitutional karyotypic abnormality (Nakamura, el at., 2001) and where a higher incidence of aneuploidy in spermatozoa retrieved from their testis may be more prone, due to the additionally impaired spermatogenesis [5].

Multiple studies have utilized the fluorescence in situ hybridization (FISH) technique to assess the genetic quality of surgically retrieved spermatozoa in comparison to their ejaculated counterpart. One of the earliest reports claimed that testicular spermatozoa present a chromosomal aneuploidy reaching 19.6%, in comparison to ejaculated spermatozoa at 13% [6]. This assessment, however, was performed on 34 men, scoring 153 to 1751 cells, and using only three chromosome probes (X, Y, 18). A later study [7], done on 27 men and assessing 98 to 1,796 spermatozoa using 4 chromosome probes, also evidenced an incidence of 11.4% aneuploidy in testicular spermatozoa compared to 2.2% in the ejaculated. In a subsequent study, the testicular sperm aneuploidy was 8.8%, still significantly higher than ejaculated sperm aneuploidy, at 0.5%. This study was carried out in 35 men, on 480 to 638 sperm cells, with 5 chromosome probes [8]. The most recent report was on 17 patients where 8 chromosomes on at least 500 sperm cells were screened. This study evidenced that testicular sperm cells presented with a higher chromosomal abnormality of 4.9% compared to 0.9% in the ejaculated sperm, however this difference did not reach statistical significance [9].

In spite of this data, the reported higher prevalence of sperm aneuploidy in testicular biopsy specimens did not translate to higher incidences of pregnancy loss or aneuploid conceptuses [10, 11]. The overall rate of congenital malformations in offspring generated from ejaculated spermatozoa was determined to be 2.6% whereas for testicular sperm, it was 2.0% [10, 12]. Prenatal diagnosis carried out on amniocentesis specimens also did not show a significant difference in the frequency of abnormal karyotypes between gestations generated with ejaculated and testicular spermatozoa [13–16]. Moreover, according to a later study, the development of children born by surgically retrieved spermatozoa did not seem to evidence any concerning outcome in relation to psychological, motorial, and overall developmental characteristics [17]. These findings were supported by other reports, which assessed pregnancy outcomes and offspring data and did not find an increased risk associated with using surgically retrieved spermatozoa as compared to ejaculated spermatozoa [18–20].

These findings persuaded us to revisit the assessment of aneuploidy in spermatozoa retrieved directly from the germinal epithelium. In an observational analysis by FISH, we compared sperm aneuploidy in ejaculated and testicular spermatozoa. We then performed this analysis in a prospective manner in a cohort of men by utilizing an enhanced FISH protocol, confirmed by the most recent molecular karyotyping technology. We also assessed the incidence of aneuploidy in men with different azoospermic origins, whether obstructive or non-obstructive. Finally, because of high DNA fragmentation in their ejaculate, we were able to measure the aneuploidy of spermatozoa retrieved from the ejaculate and their testis of the same individuals.

## Materials and methods

### Inclusion criteria and study design

Semen specimens were obtained from patients undergoing treatment for infertility at our center with their partners. Epididymal sperm was retrieved from patients undergoing microsurgical epididymal sperm aspiration due to obstructive azoospermia post vasectomy reversal, and testicular sperm was obtained from testicular biopsies in patients with non-obstructive azoospermia and hypogonadism. Donors with proven fertility served as control. The Institutional Review Board of the New York Presbyterian Hospital-Weill Cornell Medicine approved this study (IRB 1006011085), and all patients gave informed written consent to participate. In an observational study involving 93 men, FISH analysis was carried out on the spermatozoa from ejaculated and surgically retrieved specimens. To provide a confirmatory NGS assessment, a prospective analysis was executed in 22 men. In addition, a paired assessment on the ejaculated and testicular spermatozoa from the same individual was performed for three men. Aneuploidy rates were compared between the ejaculated and surgically retrieved spermatozoa, and subsequently we assessed the pregnancy outcomes with the injection of spermatozoa of different sources, controlling for maternal age.

### Spermatozoa collection and preparation

Ejaculates were provided by masturbation and evaluated according to the standards of the World Health Organization [21]. Ejaculates were centrifuged after 3:1 dilution in human tubal fluid medium (HTF Irvine Scientific, Santa Ana, CA) supplemented with HSA (HSA-Solution; Vitrolife, Sweden) at 600 g for 10 minutes to remove the seminal fluid and resuspended. Epididymal spermatozoa were obtained by epididymal aspiration and testicular spermatozoa were retrieved via testicular biopsy as previously described [22]. For FISH and TUNEL processing, specimens were smeared on a glass slide and allowed to dry overnight. When only few cells were available, individual spermatozoa were aspirated with an ICSI pipette and placed directly onto a glass slide. For NGS and ICSI, a final suspension of the specimen at 1-2x106/ml concentration was prepared.

### Preparation of spermatozoa for FISH analysis

Slides were fixed in Carnoy’s fixative (3:1 methanol:acetic acid) at room temperature for 15 minutes, and placed on a 37°C slide moat overnight. Sperm decondensation was achieved by placing slides into a Coplin jar containing 10mmol/l dithiothreitol (DTT; Sigma Chemical Co., St. Louis, MO, USA) in 100 mmol/ l tris(hydroxymetyl) aminomethane (Trizma HCl; Sigma Chemical Co.) at 22°C for 3 minutes. Slides were then washed for 1 minute in 2x standard saline citrate (SSC; Vysis, Downers Grove, IL, USA), and hybridized with fluorescent probes specific for chromosomes X, Y, 13, 15, 16, 17, 18, 21 and 22 (S1 Table). Seven ul of 4’,6-diamino-2-phenylindole (DAPI; Abbott Molecular, Des Plaines, IL, USA) was used to counterstain sperm nuclei. Each slide was then cover-slipped and assessed on a fluorescent microscope (Olympus BX61; New York/New Jersey Scientific, NJ, USA) at 1000x. Incidences of disomy, nullisomy and diploidy was assessed (S1 Fig) in at least 1000 spermatozoa for each specimen, per patient, with a threshold of >1.6% (euploid) while maintaining a 2–3% FISH error (Applied Imaging, CytoVysion v3.93.2). Slides were also processed and assessed in replicate to reduce FISH error.

### Sperm chromatin assessment

In preparation for sperm chromatin assessment, slides were fixed in 4% paraformaldehyde solution (Formaldehyde, Formalin; Electron Microscopy Sciences, Hatfield, PA, USA) for 1 hour at room temperature, then rinsed in phosphate buffered saline (PBS; Millipore Sigma, Darmstadt, Germany) three times and allowed to dry overnight. Spermatozoa were then permeabilized for 2 minutes at 4°C in 0.1% Triton X-100 (Triton X-100; Millipore Sigma, Darmstadt, Germany) and 0.1% Sodium Citrate (Sodium Citrate; Millipore Sigma, Darmstadt, Germany) in PBS. Slides were rinsed in PBS again and processed using a commercially available kit (In Situ Cell Death Detection Kit, Fluorescein; Roche, Mannheim, Germany). Sperm nuclei were counterstained with 7 ul of DAPI, cover-slipped and assessed at 1000x on a fluorescent microscope (Eclipse 50i; Nikon, Tokyo, Japan). The incidence of DNA fragmentation was assessed in at least 500 spermatozoa, with a threshold of 15% (normal).

### Whole molecular karyotype by NGS

Sperm decondensation was first carried out by incubating specimens with dithiothreitol (DTT) at 65°C for 10 minutes. DNA extraction and amplification was achieved with the use of a commercial kit (Repli-G Single Cell; Qiagen, Hilden, Germany) through PCR-based random hexamer amplification. Following amplification, DNA was submitted for quality control testing where a DNA concentration of 447.8±198ng/ul and acceptable quality of 1.7±0.1nm was confirmed for 16 specimens. These specimens were processed by Next Generation Sequencing technology and aneuploidy was assessed by recording the Copy Number Variations (CNVs). The assessment was done by comparing the study group copy number gains and losses to base-level log-ratios created from the control group. The CNVs were then ranked according to these log-ratio values.

Specimens were sent to an outside facility (Genewiz, Inc; South Plainfield, NJ, USA) where they were processed by 150-bp-paired-end sequencing on an Illumina HiSeq 2500 system, 2 samples per lane. After sequenced reads were trimmed to remove nucleotides with poor quality (error rate <0.01), they were aligned to the human reference genome (hg19) using CLC Genomics Server 9.0. Quality assessments of each indexed sample were performed by QPCR. High quality coverage (S2 Table) of 85x was obtained for each specimen (Agilent SureSelect Human All Exon V6), with at least 90% exome coverage (S2 Fig). Base calling accuracy for all samples was ~99.9%, as indicated by an average Phred quality score of Q38 (S3 Table).

After CNV detection was completed using CLC Genomics Server 9.0, the detected variants were compared with the single nucleotide polymorphism database and common variants were filtered out (S3 Fig). Homozygosity was assigned when the frequency of heterozygous single nucleotide polymorphisms was ≤5.0% (S4 Table). Remaining variants were then further annotated as being located within the coding region and used to identify gene mutations. Genes were considered duplicated when the read depth was greater than 1.5 times the median depth in the control sample, for more than 70% exons of the gene. Similarly, genes were considered deleted when the read depth was less than 0.5 times the median depth in the control sample, for more than 70% exons of the gene. All sequence data is available through the NCBI Sequence Read Archive (https://www.ncbi.nlm.nih.gov/sra) under the study accession number PRJNA489299 (S5 Table).

### Collection, preparation and evaluation of oocytes

Patient age, weight, antral follicular count, serum anti-mullerian hormone (AMH) level and previous response to stimulation were carefully examined to determine the stimulation protocol [23]. Patients were treated with daily gonadotropins (Follistim, Merck, Kenilworth, NJ, USA; Gonal-F, EMD-Serono, Geneva, Switzerland; and/or Menopur, Ferring Pharmaceuticals Inc, Parsippany, NJ, USA). Pituitary suppression was achieved with a GnRH-agonist (leuprolide acetate, Abbott Laboratories, Chicago, IL, USA) or a GnRH-antagonist (Ganirelix acetate, Merck, Kenilworth, NJ, USA; or Cetrotide, EMD-Serono Inc., Rockland, MA, USA). To attain follicular synchronization, some patients were treated with 0.1 mg estradiol patches (Climara, Bayer Healthcare Pharmaceuticals, Berlin, Germany) or oral contraceptive pills (Ortho-Novum, Janssen Pharmaceuticals, Beerse, Belgium) before starting gonadotropins. The trigger for final oocyte maturation with human chorionic gonadotropin (hCG, Ovidreal, EMD Serono) was administered when the 2 lead follicles reached a diameter of ≥17mm. Transvaginal oocyte retrieval was performed under conscious sedation 35–37 hours after hCG administration.

The oocytes retrieved were incubated for an additional 3 to 4 hours. Prior to micromanipulation, the cumulus-corona cells were removed by exposing the oocytes to medium containing 40 IU/mL of hyaluronidase (Cumulase, Halozyme Therapeutics, Inc. San Diego, CA) [24, 25]. To facilitate this process, the oocytes were aspirated in and out of a calibrated pipette stripper (Origio, Målov Denmark) with an approximate inner diameter of 200 *μm*. The complete removal of the adhering corona radiata was necessary because residual corona cells can limit visuality of the oocyte and obstruct the holding and/or injecting pipettes. Each oocyte was washed twice in culture medium (home-brew, modified Cornell medium based on G1 and G2 components) (Vitrolife, Sweden)) [26, 27] and then examined under the inverted microscope (TE2000U, Nikon USA, Melville, New York, USA) equipped with 2x, 4x, and 10x objectives (Nikon CFI Apo) and 20x and 40x objectives (Nikon Polarized optics CFI Plan Fluor) to assess integrity and maturation stage. The presence of a germinal vesicle and the absence of a polar body were signs of incomplete nuclear maturation. Following cumulus removal, oocytes at prophase I displayed a germinal vesicle, and at metaphase I the germinal vesicle breaks down (GVBD) without extrusion of the polar body (PB). Once the first PB was identifiably present, oocytes were considered at the MII stage and therefore injectable.

### ICSI procedure

The details of the injection procedure have been previously described as have the selection of the spermatozoon and the immobilization-permeabilization method [24]. Briefly, a morphologically selected spermatozoon moving in a viscous medium was injected into an MII oocyte as previously described [24]. The assessment of activation/fertilization was carried out under inverted microscope 16–18 hours after ICSI by evaluating oocytes for the presence of one or more pronuclei as well as polar bodies [24]. Oocyte activation was defined as the presence of a single pronucleus with two distinct polar bodies, while diagenic 3PN was defined as the presence of three pronuclei [28, 29]. Only oocytes with two distinct pronuclei and two clear polar bodies were considered as normally fertilized and were loaded into the incubator (EmbryoScope time-lapse system, Vitrolife) to be monitored by time lapse imaging thereafter.

### Embryo transfer and outcome assessment

Embryo transfer was carried out 3 or 5 days following microinjection. Patients received 50 mg intramuscular progesterone supplement daily, starting 24 hours after retrieval, and serum βhCG levels were then measured 14 days afterwards. Clinical pregnancy was defined as fetal heart activity detected on ultrasound.

### Statistical analysis

The two-sample *t*-test and the Mann-Whitney U test were used to compare the aneuploidy rates between ejaculated and surgically retrieved specimen, whether assessed by FISH or NGS (GraphPad Software, San Diego, CA). Friedman’s Chi-Square test (Jandel Scientific, San Rafael, CA) was used to assess pregnancy outcome generated by ejaculated or surgically retrieved spermatozoa in the different comparison groups. *P* value was reported only when it reached a 0.05 significance level.

## Results

Over a period of 4 years, a cohort of 93 couples (maternal age 35.8±4yrs and paternal age 39.4±8yrs) were included in our study. Over 90% of the couples were of White (non-Hispanic) ethnicity. Ages and semen parameters are presented in Table 1.

**Table 1:** Characteristics of non-azoospermic men and specimens screened by FISH aneuploidy.

No. of	
**Men**	87
**Age (M yrs±SD)**	39.2 ± 6
**Volume (M ml±SD)**	3.4 ± 1
**Concentration (M x10**^**6**^**/ml±SD)**	39.8 ± 34
**Motility (M%±SD)**	23.0 ± 26
**Morphology (M%±SD)**	1.5 ± 2

**Fig 1:**
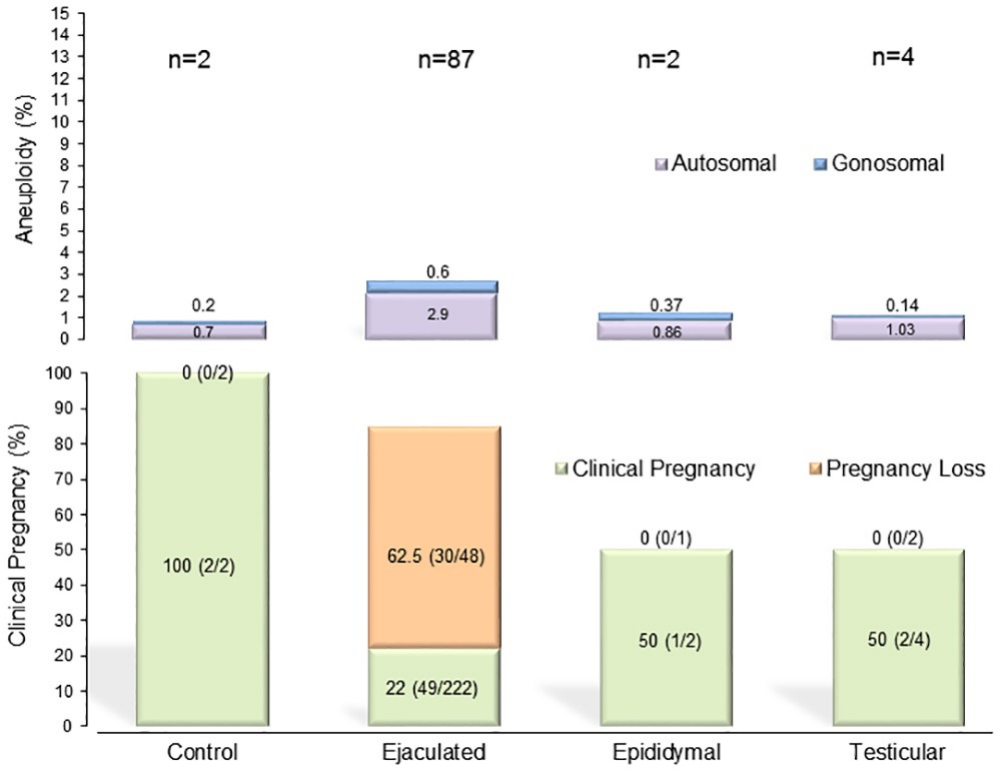
Sperm aneuploidy assessed by FISH in specimens of different origins with related pregnancy outcome. The two-sample *t*-test and the Mann-Whitney U test were used to compare the autosomal and gonosomal aneuploidy rates assessed by FISH in at least 1000 spermatozoa of ejaculated (n = 87), testicular (n = 4), and epididymal (n = 2) specimens in comparison to the donor control group (n = 2) (Upper graph). Friedman’s Chi-square test was then used to compare the clinical pregnancies and their evolution into miscarriage or term. These outcomes are represented according to the source of spermatozoa used for ICSI (Lower graph). No significant differences were found in both statistical comparisons.

FISH analysis was performed to assess the 9 chromosomes that were most clinically relevant [30–32]. For each patient, the multiple incidences of disomy, nullisomy, and diploidy were evaluated in at least 1000 spermatozoa. This assessment revealed an overall aneuploidy of 3.6% in men who provided ejaculated specimens (n = 87), 1.2% in the men who provided epididymal specimens (n = 2), and 1.1% in the men who provided testicular specimens (n = 4), compared to 0.9% in the donor specimens serving as a control (Fig 1, S6 Table). No significant differences were found when the two-sample t-test and Mann-Whitney U test were used to compare autosomal or gonosomal disomies as well as nullisomies. The injection of the ejaculated spermatozoa yielded a 22% (49/222) clinical pregnancy rate that ended in 62.5% (30/48) miscarriages, while the surgical specimens yielded a 50% (1/2) clinical pregnancy rate that progressed to term (Fig 1).

To confirm these findings, a Copy Number Variation (CNV) assessment was performed in a prospective manner on the male partner specimens of 22 couples (maternal age 37.7±1yrs and paternal age 38.3±7yrs), using NGS. Age and sperm characteristics are presented in Table 2.

**Table 2:** Characteristics of couples and semen specimens screened by NGS aneuploidy.

***Participants***	
**Men**	16
**Male age (M yrs±SD)**	38.3 ± 7
**Female age (M yrs±SD)**	37.7±1
***Semen Parameters***	
**Concentration (M x10**^**6**^**/ml±SD)**	29.6 ± 33
**Motility (M%±SD)**	24.6 ± 25
**Morphology (M%±SD)**	1.6 ± 2

**Fig 2:**
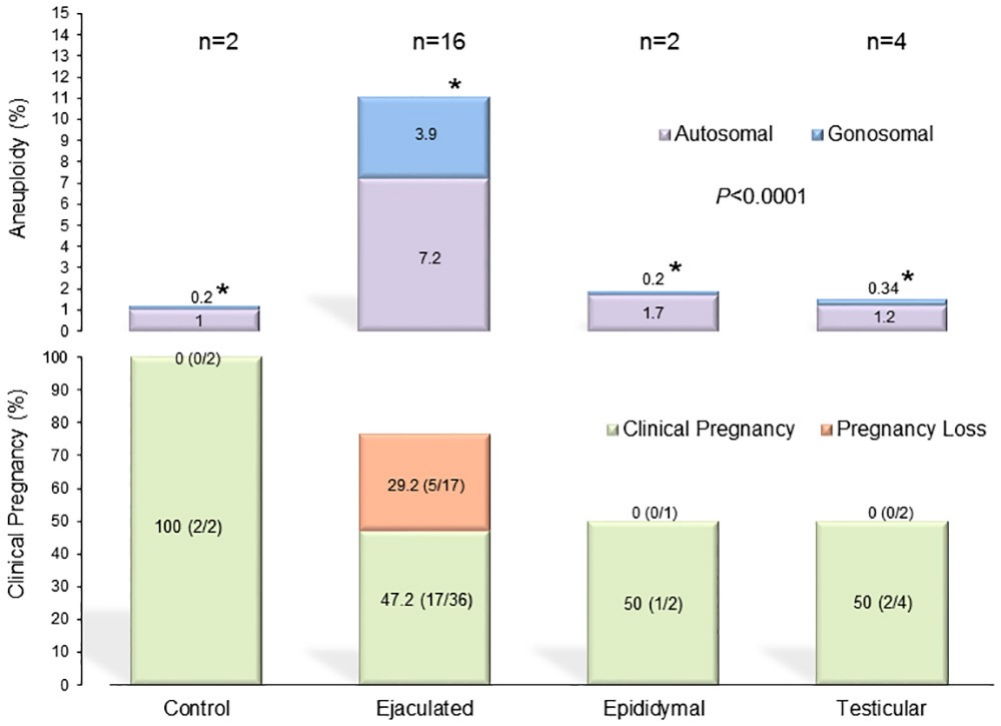
Sperm aneuploidy assessed by NGS in specimens of different origins with related pregnancy outcome. The two-sample *t*-test and Mann-Whitney U test were used to compare the autosomal and gonosomal aneuploidy rates assessed by NGS in at least 1000 spermatozoa and evidenced that the ejaculated (n = 16) specimen had a higher incidence of aneuploidy than the epididymal (n = 2), testicular (n = 4), and donor control specimen (n = 2) (*P*<0.0001) (Upper graph). In the lower graph, the clinical pregnancies and their evolution into miscarriage or term, assessed by Friedman’s Chi-square test, are represented according to the source of spermatozoa used for ICSI. No statistical difference was found in this analysis.

Interestingly, NGS yielded a total aneuploidy of 11.1% in the ejaculated group (n = 16), that decreased to 1.8% in the epididymal (n = 2) and remained at 1.5% for the testicular group (n = 4) (*P*<0.0001) (Fig 2). The pregnancy rate following the injection of the ejaculated specimen was 47.2% (17/36) with 29.2% (5/17) pregnancy loss and for the surgically retrieved, 50% that progressed to term (Fig 2). In order to measure the aneuploidy occurrence in relation to germinal epithelium function on spermatogenic progression, an aneuploidy assessment by NGS was also carried out among a subgroup of these men who underwent exclusively testicular retrieval, which according to the indication of their azoospermia, evidenced 6.7% aneuploidy in the obstructive (n = 3) and 5.1% in the non-obstructive men (n = 3) (S7 Table).

**Fig 3:**
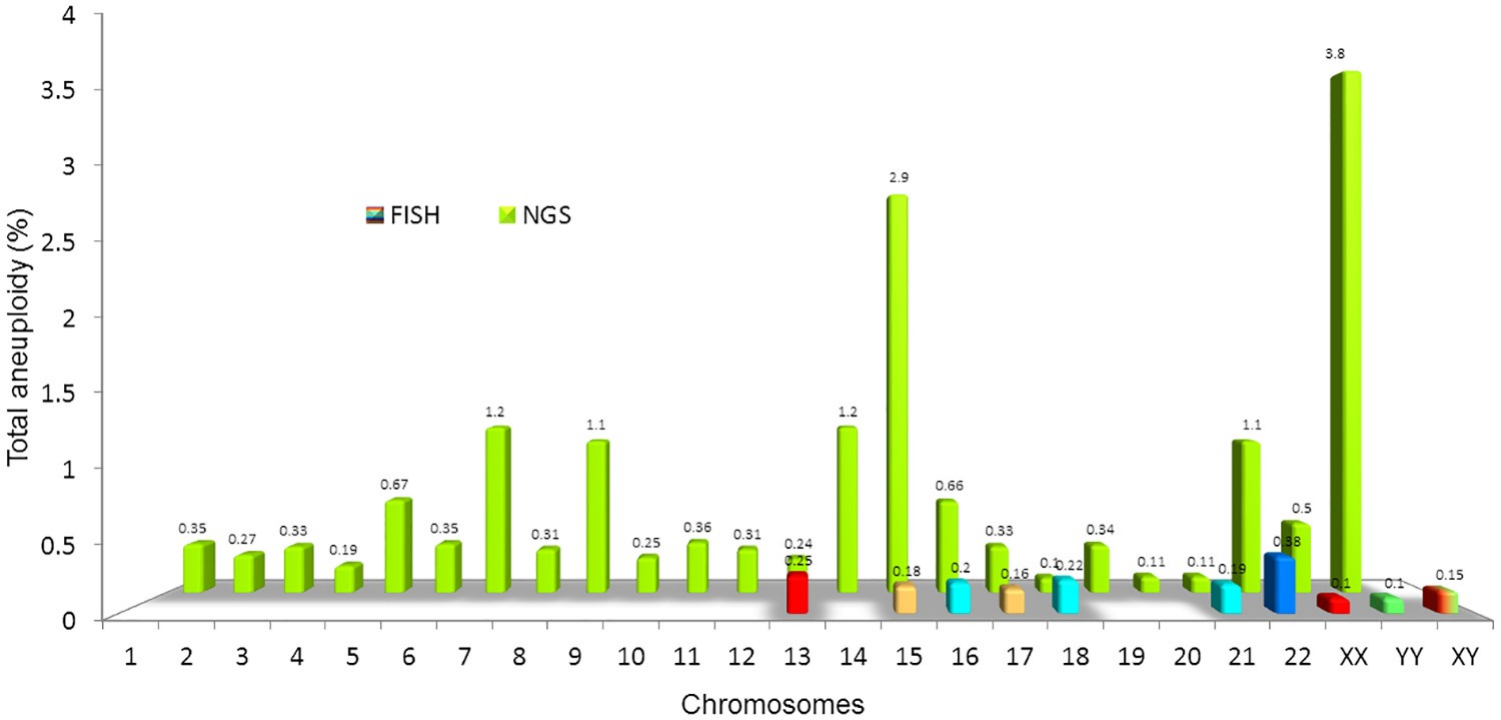
Aneuploidy comparison per chromosome by FISH and NGS. Aneuploidy assessment by FISH, when compared to NGS, appears minute. This dramatic difference between the two assays demonstrates the sensitivity of the NGS assessment.

Aneuploidy and CNV results, assessed by FISH and NGS respectively, were also compared in a sub-analysis. We found that for each chromosome assessed by FISH, the incidence of aneuploidy appeared minute when compared to CNV occurrences identified using NGS. The most dramatic differences were observed for chromosomes 15 and Y, in which FISH yielded 0.18% aneuploidy for chromosome 15 and 0.1% for chromosome Y, while NGS yielded 2.9% and 3.8% aneuploidy, respectively (Fig 3).

An additional prospective assessment carried out by FISH and NGS was performed for 3 men who underwent testicular biopsies, in spite of having a normal spermatogenesis. Age and sperm characteristics are presented in Table 3.

**Table 3:** Characteristics of sperm specimens from different origin obtained from the same men and screened by FISH and NGS.

***Participants***		
**Men**	3	
**Male age (M yrs±SD)**	35.2 ± 1.1	
***Semen Parameters***	**Ejaculate**	**Surgically Retrieved**
**Concentration (M x10**^**6**^**/ml±SD)**	16.3 ± 27	2.0 ± 3
**Motility (M%±SD)**	22 ± 15	0.5 ± 1
**Morphology (M%±SD)**	1.7 ± 1	--

**Fig 4:**
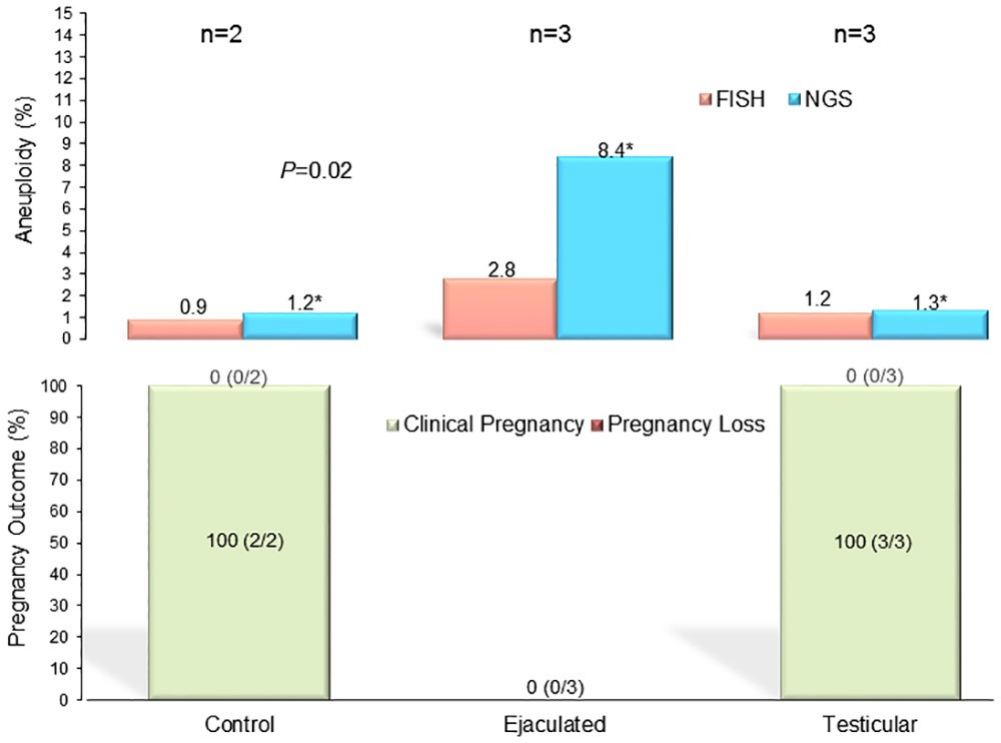
Sperm aneuploidy assessed by FISH and NGS of ejaculated and testicular specimens from the same individuals with related pregnancy outcome. The two-sample *t*-test and Mann-Whitney U test were used to compare the autosomal and gonosomal aneuploidy rates assessed concurrently by FISH and NGS, in the same individuals, on at least 1000 spermatozoa. A higher incidence of aneuploidy was found in the ejaculated (n = 3) in comparison to the testicular (n = 3) and donor control specimens (n = 2) (*P* = 0.02) (Upper graph). In the lower graph, the Friedman's Chi-square used to compare the clinical pregnancy according to the source of spermatozoa used for ICSI did not evidence any statistical difference.

In these patients, testicular biopsy was justified by the high DNA fragmentation in their ejaculated specimens and required a thorough counseling by the reproductive urologist prior to the procedure. These men had an average sperm chromatin fragmentation of 38% in the ejaculate spermatozoa that resulted to be 8% in their testicular spermatozoa (*P*<0.001). When FISH technique was used, the ejaculate specimens had an aneuploidy rate of 2.8% while the testicular specimens had a 1.2% overall aneuploidy. This trend was corroborated by an NGS assessment, which yielded 8.4% aneuploidy in the ejaculate and 1.3% aneuploidy in the testicular spermatozoa (*P* = 0.02) (Fig 4).

Interestingly, ICSI cycles in these couples who underwent simultaneous assessment by FISH and NGS using ejaculated spermatozoa resulted in no (0/3) clinical pregnancies while the cycles using testicular spermatozoa resulted in a 100% (3/3) clinical pregnancy rate, all to term (Fig 4).

## Discussion

The contribution of the paternal genome to the aneuploidy of the conceptus has generally been considered negligible. However, this information has been gaining renewed interest, with the utilization of testicular spermatozoa retrieved from men with azoospermia. This is particularly relevant because these individuals have a higher incidence of peripheral karyotype aneuploidy [33] and present significantly higher chromosomal defects on the spermatozoa retrieved from their testicle [6]. Surprisingly, the increasing popularity of retrieving spermatozoa directly from the seminiferous tubule has not been accompanied by a higher incidence of miscarriages or neonatal chromosomal abnormalities.

In this study we utilized an improved FISH technique that screens a greater number of chromosomes that, in contradiction to previous studies [6–8], aims at reassessing the aneuploidy of testicular spermatozoa. We carried out our FISH analysis on the 9 chromosomes that are most clinically relevant according to previous studies [30–32]. To our dismay, in this study we observed that surgically retrieved spermatozoa possessed a comparable aneuploidy rate than ejaculated spermatozoa. FISH, however, is known to imply several limitations such as hybridization failure and low fluorescent response due to insufficient permeabilization of the cell membrane, resulting in FISH error [34].

We therefore challenged the FISH technique by carrying out the same assessment in another group of men using NGS. The use of Next Generation Sequencing has gained increased attention due to its ability to assess the entire chromosome array while not being limited by the accessible number of cells [35–37]. Our prospective NGS testing of sperm chromosomes also failed to evidence a surge in the occurrence of chromosomal imbalances in testicular spermatozoa of azoospermic men when compared to men who had screened their ejaculated gametes. The utilization of these men’s spermatozoa to inseminate the oocytes of their partner resulted in a comparable pregnancy rate than that of men who provided ejaculated spermatozoa.

We then questioned whether among azoospermic men, the degree of spermatogenic function would have an effect on the meiotic mechanism and therefore provide higher chances of chromosomal imbalance. Indeed, in a subset of men with non-obstructive azoospermia and compromised spermatogenesis, NGS testing showed similar levels of aneuploidy when compared to men with obstructed azoospermia [38].

We also decided to determine if the maturation of the male gamete achieved through the progression within the genital tract, putatively because of a corrective role exerted by the epididymis, would have any effect on aneuploidy occurrence. Thus, we assessed aneuploidy on ejaculated and testicular spermatozoa, concurrently by FISH and NGS, in the same individuals. This was feasible because these men had a high DNA fragmentation in their ejaculate and preliminary studies have evidenced a lower SCF in spermatozoa retrieved directly from the seminiferous tubules [39]. In this analysis, we did observe a similar aneuploidy rate in testicular spermatozoa; indeed it was lower (*P*<0.0001). In support of these findings, the pregnancy rate appeared comparable and even higher in couples where surgically retrieved spermatozoa for ICSI were used on the female partners’ oocytes.

In concordance with previous reports, this study suggests that testicular spermatozoa in combination with ICSI is an effective and safe method of treatment for severe male infertility [40–44].

These unexpected findings appear to contradict earlier concerns related to the high chromosomal defects in spermatozoa retrieved from the testicle. An analogy may be made with the level of SCF that appears to be lower in testicular spermatozoa than in the ejaculate [42, 45–48]. While sperm DNA fragmentation appearance has been attributed to the action of oxidative stress exerted within the male genital tract, the occurrence of higher aneuploidy in the ejaculated spermatozoa may be due to a defect in epididymal phagocytotic function, capable of sequestering spermatozoa in the cauda without reabsorbing them [49]. These residual spermatozoa would then be leaked during ejaculation, yielding the apparently higher proportion of aneuploidy in the semen sample. In healthy individuals, these spermatozoa may be easily identified and destroyed within the epididymal epithelium by a ubiquitin-dependent mechanism, as reported in the mouse [50], which may explain the low aneuploidy rates of ejaculated spermatozoa in healthy donors.

These are preliminary findings, and they appear very promising. However, we recognized that the study is carried out on a limited number of subjects. If confirmed by independent investigators, these findings may indicate that the meiotic mechanism responsible for the generation of euploid gametes is not affected by the proportion of cells that progress through the spermatogenic line, indicating that it is safe to use testicular spermatozoa at least in regard to their chromosomal content.

## Conclusions

In light of the availability of a more accurate molecular genetic technique, namely NGS, we revisited the notion that epididymal and testicular tissues yield spermatozoa with a higher incidence of aneuploidy as compared to those retrieved from the ejaculate. The findings of this study have shown that the total aneuploidy of surgically retrieved spermatozoa is certainly comparable to that of ejaculated spermatozoa. This may explain why pregnancies resulting from the injection of testicular gametes isolated from azoospermic men are not at a higher risk of miscarriage and the resulting offspring do not show a higher autosomal or gonosomal aneuploidy than the children resulting from ejaculated spermatozoa.

## Supporting information

S1 Table FISH probes. List of FISH probes, signal for each, probe name and locations.
(PDF)

S2 Table Details on NGS coverage. NGS coverage details including target regions and length of coverage for each sample.
(XLSX)

S3 Table WES quality metrics. Quality metrics for each sample, including total and captured reads and coverage.
(XLSX)

S4 Table Polymorphic CNV assessment. Assessment for polymorphic CNVs performed using the Database for Genomic Variants.
(XLSX)

S5 Table FASTQ accession numbers. NCBI accession numbers of NGS FASTQ files for each individual specimen.
(XLSX)

S6 Table FISH results for each patient. FISH aneuploidy results for each chromosome assessed, per patient.
(XLSX)

S7 Table CNV working results. Working results for CNV analysis.
(XLSX)

S1 Fig FISH image. Images of positive FISH signals.
(TIF)

S2 Fig NGS sample prep workflow. Workflow illustrating specimen prep and sequencing.
(TIF)

S3 Fig NGS bioinformatics workflow. Workflow illustrating how raw FASTQ data were processed to assess CNV.
(TIF)

S4 Fig Results scheme workflow. Graphical scheme for study results with key findings.
(TIF)

S1 File VCF files. VCF files containing CNV analysis for each specimen.
(ZIP)
