# Supplementary material for: Revisiting aneuploidy profile of surgically retrieved spermatozoa by whole exome sequencing molecular karyotype
Source: PLoS One. 2019 Jan 4;14(1):e0210079. doi: 10.1371/journal.pone.0210079 (PMC6319716; doi:10.1371/journal.pone.0210079)
Supplement: S1 Table — List of FISH probes, signal for each, probe name and locations. (PDF) [file pone.0210079.s001.pdf]

S1 Table. List of FISH probe Sets, probe locations and fluorophore signals for each

Oligo X Y 15 17 (OF4-0052-0100)		
**Chromosome**	**Location**	**Fluorophore**
X	Centromere, locus DXZ1	Red
Y	Yq12 Satellite III DNA	Green
15	Centromore, locus D15Z1	Gold
17	Centromere, locus D17Z1	Aqua
**Oligo X Y 16 18 (OF4-0135-0100)**		
**Chromosome**	**Location**	**Fluorophore**
X	Centromere, locus DXZ1	Red
Y	Yq12, locus DYZ1	Gold
16	Centromere, locus D16Z1	Green
18	Centromere, locus D18Z1	Aqua
**Vysis MultiVysion PGT Multi-color Probe (05J32-080)**		
**Probe**	**Location**	**Fluorophore**
Vysis LSI 13	13q14	Red
Vysis LSI 21	21q22.13-22.2	Green
Vysis CEP 18	18p11.1-q11.1 Alpha Satellite DNA	Aqua
Vysis CEP X	Xp11.1-q11.1 Alpha Satellite DNA	Blue
Vysis CEP Y	Yp11.1-q11.1 Alpha Satellite DNA	Gold
**Vysis MultiVysion PB Multi-color Probe (05J31-085)**		
**Probe**	**Location**	**Fluorophore**
Vysis LSI 13	13q14	Red
Vysis LSI 21	21q22.13-22.2	Green
Vysis LSI 22	22q11.2	Gold
Vysis CEP 16	16q11.2 Satellite II DNA	Aqua
Vysis CEP 18	18p11.1-q11.1 Alpha Satellite DNA	Blue
