# Supplementary figures and images for: Revisiting aneuploidy profile of surgically retrieved spermatozoa by whole exome sequencing molecular karyotype
Source: PLoS One. 2019 Jan 4;14(1):e0210079. doi: 10.1371/journal.pone.0210079 (PMC6319716; doi:10.1371/journal.pone.0210079)

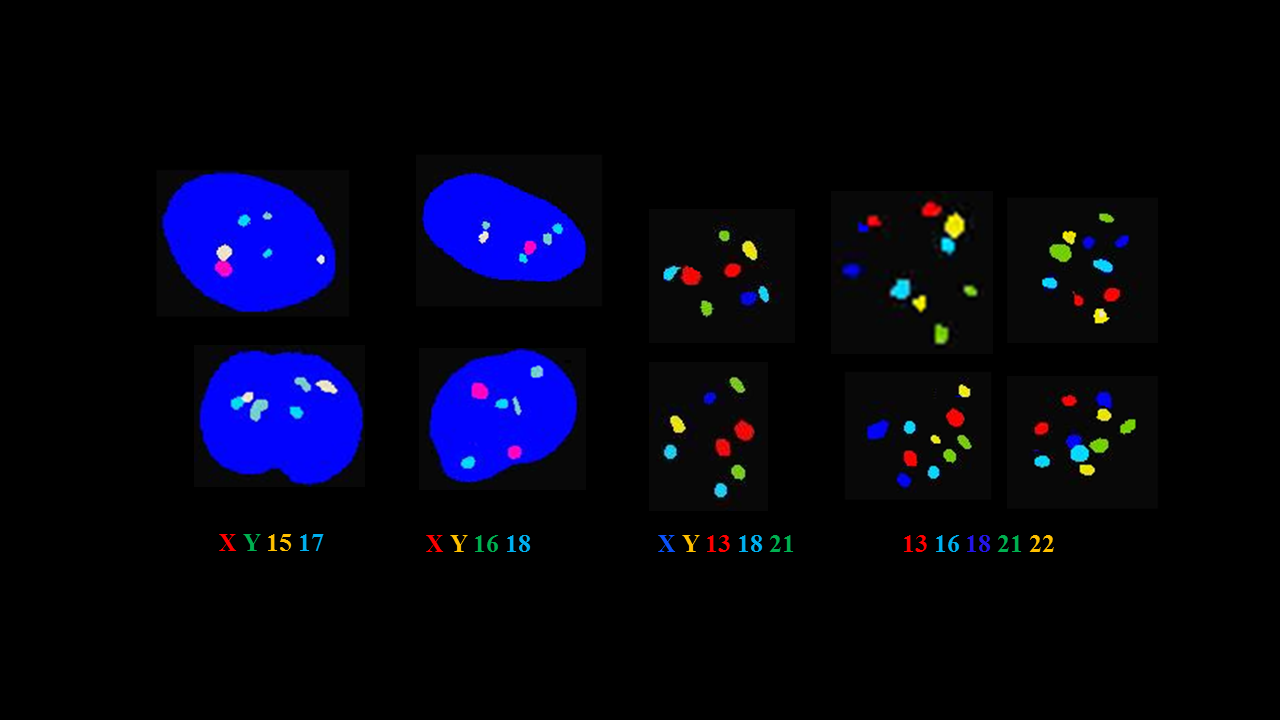

Supplement: S1 Fig — Images of positive FISH signals. (TIF) [file pone.0210079.s008.tif]

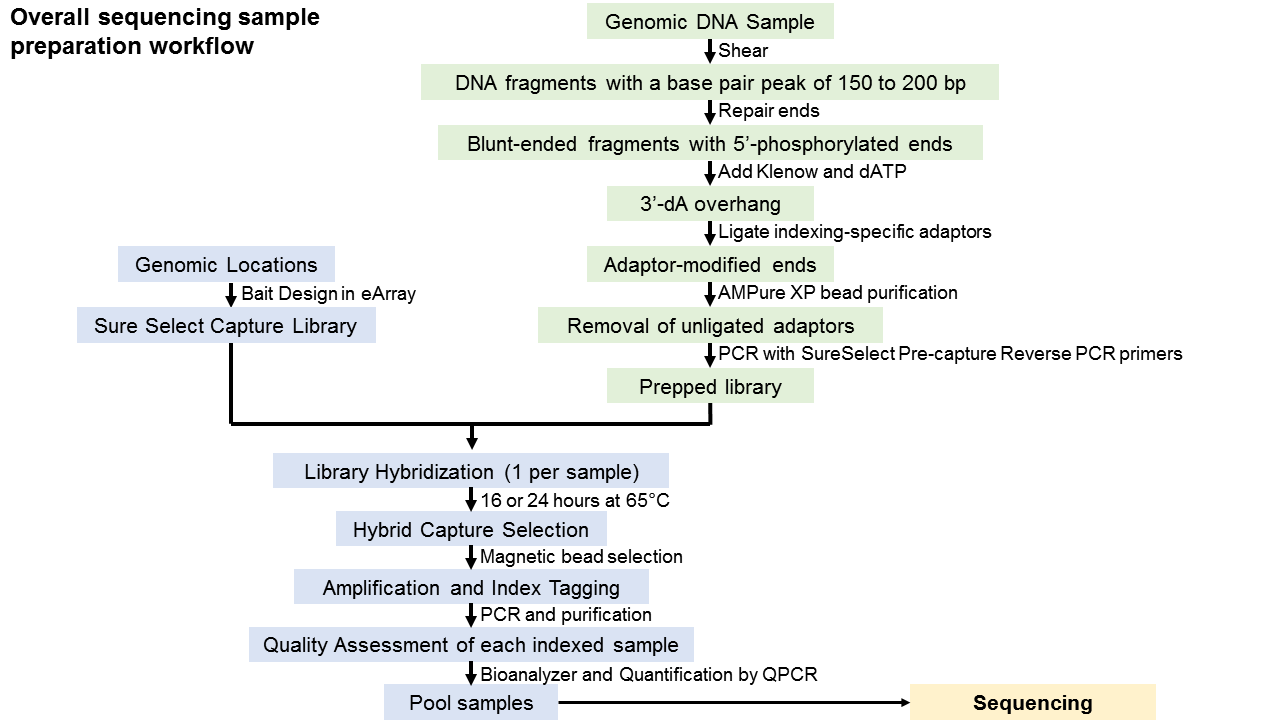

Supplement: S2 Fig — Workflow illustrating specimen prep and sequencing. (TIF) [file pone.0210079.s009.tif]

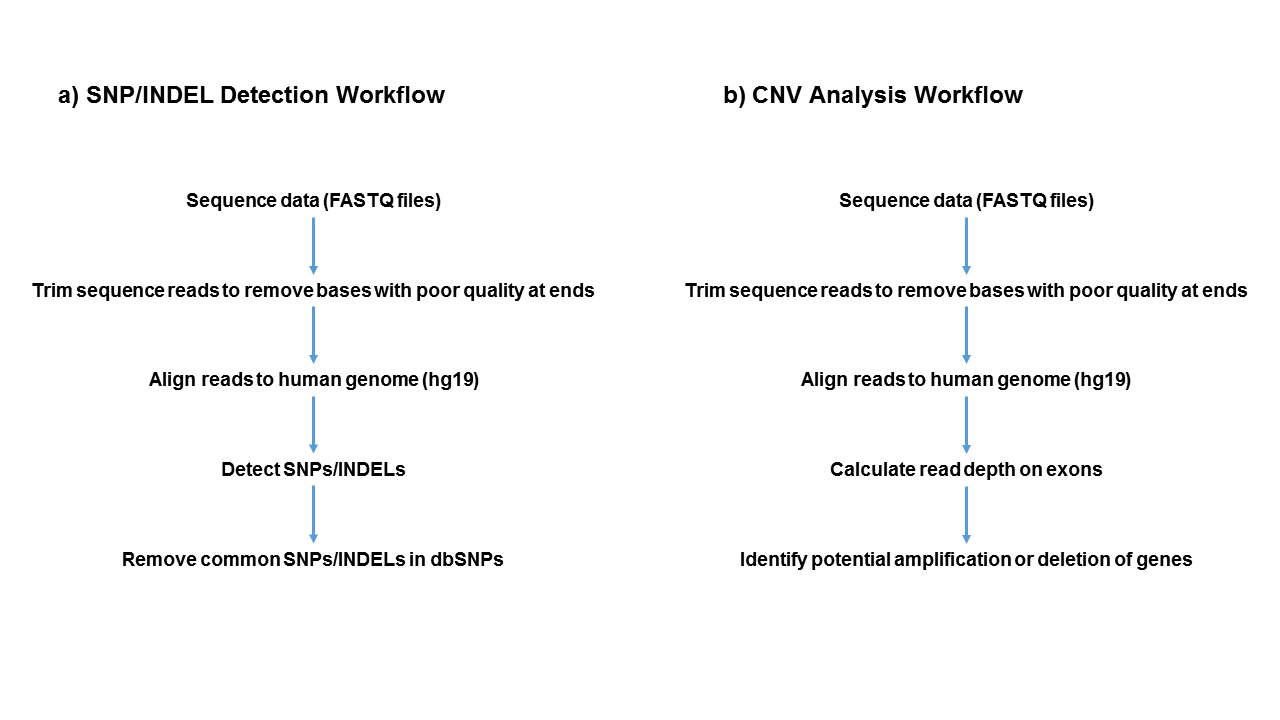

Supplement: S3 Fig — Workflow illustrating how raw FASTQ data were processed to assess CNV. (TIF) [file pone.0210079.s010.tif]

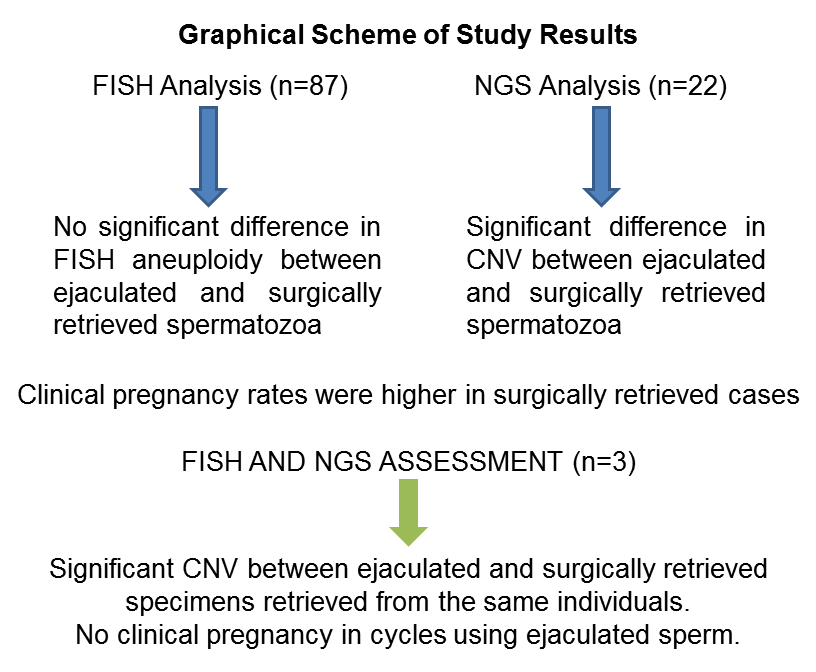

Supplement: S4 Fig — Graphical scheme for study results with key findings. (TIF) [file pone.0210079.s011.tif]
